# Targeting tumor angiogenesis with TSP-1-based compounds: rational design of antiangiogenic mimetics of endogenous inhibitors

**DOI:** 10.18632/oncotarget.200

**Published:** 2010-11-25

**Authors:** Giulia Taraboletti, Marco Rusnati, Laura Ragona, Giorgio Colombo

**Affiliations:** ^1^ Department of Oncology, Mario Negri Institute for Pharmacological Research, Bergamo, Italy; ^2^ Department of Biomedical Sciences and Biotechnology, School of Medicine, University of Brescia, Brescia, Italy; ^3^ Istituto per lo Studio delle Macromolecole, Consiglio Nazionale delle Ricerche, Milan, Italy; ^4^ Istituto di Chimica del Riconoscimento Molecolare, Consiglio Nazionale delle Ricerche, Milan, Italy

**Keywords:** tumor, oncotarget, angiogenesis, TSP-1

## Abstract

Inhibitors of angiogenesis are an important addition to conventional chemotherapy. Among different “druggable” angiogenic factors, fibroblast growth factor-2 (FGF-2) is an attractive target for novel therapies because of its intricated involvement in tumor neovascularization, tumor cell proliferation and migration, and the acquisition of resistance to antiangiogenic therapies. FGF-2 bioavailability and activity is affected by several natural ligands, including the endogenous inhibitor of angiogenesis thrombospondin-1 (TSP-1). We hypothesized that the FGF-2-binding sequence of TSP-1 might serve as a template for the development of non-peptide inhibitors of angiogenesis. Computational biology and nuclear magnetic resonance spectroscopy approaches, major investigative tools in the characterizations of protein-protein interaction (PPI), were used to map the residues at the TSP-1/FGF-2 interface. The translation of this three-dimensional information into a pharmacophore model allowed screening a small molecule databases, identifying three FGF-2-binding, antiangiogenic small molecules, mimetic of TSP-1. Pharmacophore-based approaches are thus feasible tools to exploit naturally occurring PPI, by generating a set of lead compounds mimetic of endogenous proteins, as a starting point for the development of novel therapeutic agents.

## INTRODUCTION

Angiogenesis has become a successful target in cancer therapy [1]. Designed to target the formation of a functional vascular network – a requirement for the malignant progression -, antiangiogenic agents impair tumor growth and metastatic dissemination [2]. These drugs, mostly inhibitors of the angiogenic factor vascular endothelial growth factor (VEGF), have become important tools in the clinical practice, usually in combination with conventional chemotherapy. However, antiangiogenic therapies still cause only a modest increment of overall survival, and often present relevant toxic effects. The lack of long-lasting therapeutic effects of the antiangiogenic therapies in neoplastic patients is due to acquired (“evasive”) resistance to these agents resulting from a concurrence of causes including tumor adaptation to growth in an angiogenesis-independent manner, selection of more malignant and invasive tumor cells by therapy-induced hypoxia, and increased production of angiogenic factors, equal and/or different from the targeted one [3]. Several approaches have been proposed to overcome resistance. The optimization of schedule of administration and length of treatment with the antiangiogenic agents is certainly a relevant issue. In addition, the simultaneous targeting of different angiogenesis pathways is another possible approach to overcome the arising of resistance. So far, the antiangiogenic agents approved for clinical use target (exclusively or preferentially) VEGF. The design of agents targeting other angiogenic factors is becoming a promising field for the development of novel antiangiogenic compounds, further supported by the evidence of selective, non-redundant roles of the different angiogenic factors produced by tumors in promoting not only tumor angiogenesis and metastasis, but also the direct growth and invasion of tumor cells [4]. Therefore each angiogenic factor represents an important target for therapy of tumors, challenged or not with antiangiogenic therapies.

## ANGIOGENIC GROWTH FACTORS AS TARGETS: THE PROTOTYPE FGF-2

Numerous inducers of angiogenesis have been identified, including the members of the already mentioned VEGF family, hepatocyte growth factor (HGF), angiopoietins, transforming growth factor-α and -β (TGF-α and -β), platelet-derived growth factor (PDGF), tumor necrosis factor-α (TNF-α), interleukins, chemokines, and the members of the fibroblast growth factor (FGF) family [1,2,5].

Beside VEGFs, FGFs are recognized targets for the development of anti-cancer therapy [6,7]. FGF-2 has been the first tumor-associated angiogenic factor to be purified [8]. Since then, an increasing amount of evidence has accumulated supporting the involvement of FGFs in different steps of cancer progression. Overexpression or genetic alterations lead to a deregulated activation of FGF/FGF receptor pathways in cancer [7]. Plasma levels of FGFs are frequently elevated in cancer patients, in some cases associated with tumor escape from antiangiogenic therapy [9]. Evidences indicate that FGFs, produced by both tumor or host cells, promote tumor progression both directly, by affecting tumor cell differentiation, proliferation, survival, invasion, metastasis, response to chemotherapy and cancer stem cell self-renewal, and indirectly, by inducing angiogenesis as well as the recruitment and activation of tumor-supporting stromal cells [6,7]. Therefore targeting FGFs has a multivalent value as a way to simultaneously affect different pathways associated with both tumor progression, angiogenesis, host cells recruitment and tumor resistance.

At present, 22 structurally-related members of the FGF family have been identified, including 18 FGFs (defined as FGF receptor ligands) and 4 FGF-homologous factors [6,7,10]. FGFs are pleiotropic factors that act on different cell types in autocrine, paracrine of juxtacrine manners, through different receptors, including tyrosine kinase (TK) receptors (FGFRs), heparan-sulfate proteoglycans (HSPGs), integrins, and gangliosides. Among the paracrine FGFs, FGF-1, 2, 4, 5 and 8 have pro-angiogenic activity [11].

**Figure 1:**
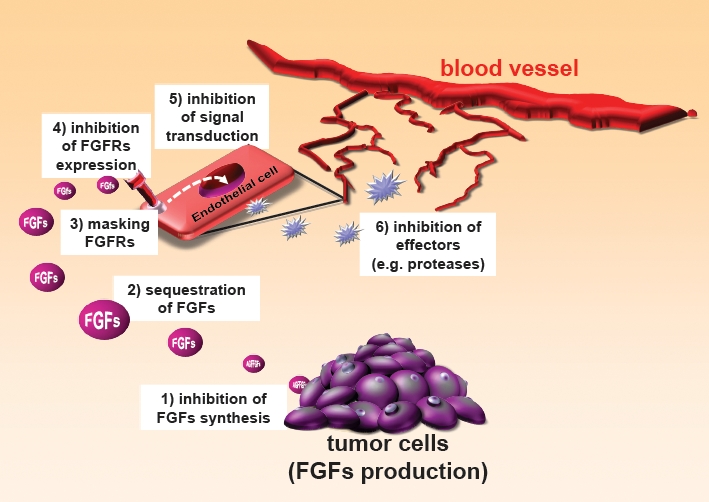
Strategies for inhibiting FGFs Inhibitors of FGFs can act by reducing FGF production by the tumor (1), interfering with FGF-FGFR recognition (2,3), affecting endothelial cells expression of FGFR (4), inhibiting FGF-induced intracellular signalling pathways (5), or act downstream FGFs, on effectors of angiogenesis (6).

The angiogenic activity of FGFs can be neutralized by different strategies, schematized in Figure 1. For a detailed review see [6,7,12].

Inhibition of FGFs production/release by FGFs producing cells (leukocytes, tumor, and stromal cells) can been achieved by antisense or dominant negative cDNAs approaches. Interestingly, chemotherapeutics have been demonstrated to inhibit FGF production, mainly by affecting FGF-producing tumor cells.

Once produced and released, FGFs can be sequestered in the extracellular space preventing their paracrine or autocrine stimulation of target cells. This can be achieved with anti-FGFs antibodies or with FGF traps, designed to mimic FGF-sequestering molecules. The finding that the released, soluble extracellular portion of the FGFR1 binds FGF-2 and prevents FGF-2/FGFR1 interaction has led to the design of FP-1039, a soluble fusion protein that consists of extracellular FGFR1-IIIc domain fused to the Fc domain of IgG1.

Heparin, a negatively charged glycosaminoglycan released in the blood stream during inflammation, sequesters FGFs in the extracellular environment, efficiently competing for the binding of the growth factor to HSPGs. Although it exerts a potent FGF-antagonist effect, unmodified heparin cannot be used as an anti-angiogenic drug because of its anticoagulant activity and binding to a wide array of physiological molecules. This prompted the development of synthetic heparin derivatives and heparin-like molecules (such as the prototypic suramin) endowed with a more specific FGF-antagonist activity and a more favorable therapeutic window (reviewed in [12,13]). Among structurally different derivatives, Gentisic acid (2,5-dihydroxybenzoic acid), a widespread plant secondary metabolite and a catabolite of aspirin, binds the heparin-binding site of FGF and has been used as a template to develop inhibitors of angiogenesis [14].

FGF receptors and ligands (including FGFRs, HSPGs, integrins and gangliosides) are important targets to block FGFs activity. The binding of FGFs to their FGFRs can be neutralized by specific antibodies directed against the receptors or by protamine or synthetic peptides corresponding to the receptor recognition sequences of FGFs. HSPGs can be masked by protamine, histidine-rich glycoprotein, PF4, endostatin and kallistatin. Integrins-FGF interactions are masked by synthetic peptides, peptidomimetics and disintegrins (a class of naturally occurring integrin antagonists) containing integrin-recognition motifs such as RGD. Finally, the cholera toxin B subunit binds to cell surface GM_1_ ganglioside hampering its interaction with FGF-2 thus inhibiting FGF-2-dependent pro-angiogenic activation of endothelial cells.

The blockage of FGF activity can be achieved by hampering the expression of the various FGF receptors on target cells, including FGFRs, HSPGs, integrins, and gangliosides. FGFRs expression can be reduced by antisense cDNA, the synthetic retinoid fenretinide, and antibodies directed against α_v_β_3_ and α_5_β_1_ integrins. Perlecan antisense cDNA and lead exposure cause HSPG downregulation. Finally, inhibitors of the synthesis of complex gangliosides, including fumonisin B_1_, D-threo-1-phenyl-2-decanoylamino-3-morpholino-1-propanol, and D-1-threo-1-phenyl-2-hexadecanoylamino-3-pyrrolidino-1-propanol, inhibit EC proliferation triggered by FGF-2.

The TK activity of angiogenic factor receptors is a successful strategy to affect angiogenesis, as shown by the number of TK inhibitors that have been subjected to clinical trials or approved in the last decade. Most TK inhibitors have a broad-spectrum activity, affecting multiple receptors and pathways, as in the case of SU5402, that targets both VEGFR-2 and FGFR1. Efforts are currently ongoing to design TK inhibitors selective for the FGFRs, such as PD173074, selective for FGFR1 and FGFR3 [6,7]. Recently, structure-guided approaches are being used to introduce focused structural changes and optimize potency and selectivity of TK inhibitors [15].

Intracellular signals activated by FGFs in tumor cells or ECs might be considered targets for angiogenesis inhibitors. A long list of synthetic compounds, dominant negative mutants or antisense cDNAs targeting FGFs-dependent signalling pathways has been described. Finally, the “pro-angiogenic phenotype” induced by FGF-2 in endothelial cells, can be targeted by agents preventing FGF-induced invasion, motility, matrix degradation, proliferation and survival.

## ENDOGENOUS INHIBITORS AS TEMPLATES TO DESIGN ANTIANGIOGENIC AGENTS: TSP-1

Endogenous inhibitors of angiogenesis have evolved as the optimal physiological control of the angiogenic process. It is therefore logical to consider them as models for the design of antiangiogenic therapies [16]. This heterogeneous group of molecules includes proteins, polysaccharides and glycosphingolipids found in the body fluids and ECM.

Thrombospondin-1 (TSP-1) was the first endogenous inhibitor of angiogenesis to be identified [17,18]. Of the 5 members that compose the TSP family in mammals, TSP-1 and TSP-2 (forming the group A, homotrimeric TSPs) are similar in domain organization and share the ability to inhibit angiogenesis. TSPs are modular proteins. Each TSP-1 monomer consists of an N-terminal globular domain, followed by the coiled-coil oligomerization domain, a von Willebrand Factor type C, procollagen domain, three properdin-like type I repeats, and a signature domain comprising three epidermal growth factor (EGF)-like type II repeats, a calcium-binding wire - type III repeats, and the lectin-like C-terminal globular domain [19].

**Figure 2:**
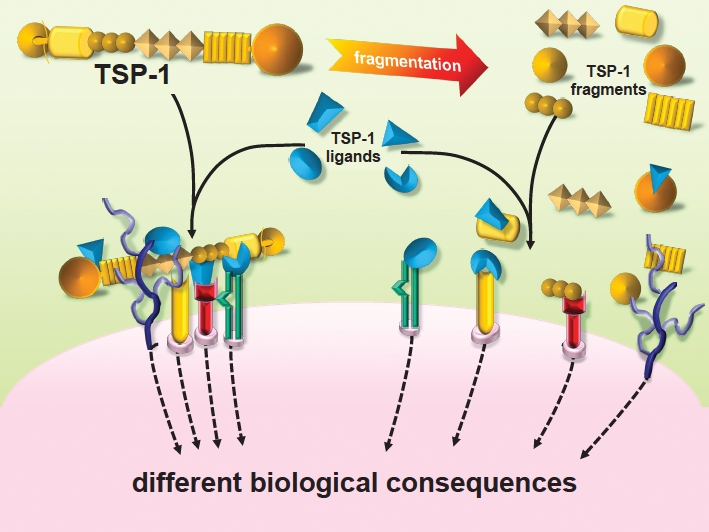
TSP-1 affects cell response to the environment: different effects of the intact molecule vs proteolytic fragments

TSP-1 is a matricellular protein, i.e. a non-structural extracellular protein that acts to regulate cell interactions with the environment [20]. Through its different domains, TSP-1 is able to interact simultaneously with different cell receptors, soluble cytokines and growth factors, extracellular matrix components, and proteases. This accounts for the pleiotropic nature of TSP-1, which, depending on the environmental properties (presence of receptors, ligands, proteases) can have different effects - even opposite - on cell activities and biological process [21-23]. Such a large, modular protein has the potential to aggregate molecular complexes at the pericellular level, acting as a scaffold that place in close proximity soluble molecules, growth factors, proteases and recruited cell receptors, ultimately orchestrating a complex signaling cascade within the cells (Figure 2). On the other hand, proteolytic digestion of TSP-1 generates active fragments which are still able to interact with their ligands, without however generating large molecular complexes, and hence with functional consequences different from entire TSP (Figure 2). The generation of antiangiogenic fragment of TSP-1 by proteases has been described [24], and indeed, proteolytic degradation of large molecules is emerging as an important mechanism to generate inhibitors of angiogenesis [25]. In the case of TSPs, it can be anticipated that antiangiogenic fragments might be characterized and taken as models for the design of inhibitors of angiogenesis.

TSP-1 can inhibit angiogenesis both directly and indirectly [26]. As a direct inhibitor it interacts with specific receptors on endothelial cells (including CD36, CD47, integrins, HSPG, and LRP) to affect cell viability and functions related to angiogenesis. Besides endothelial cells, TSP-1 is also active on other cell types involved in the angiogenic process, including smooth muscle cells, monocytes/macrophages and T cells. Finally, TSP-1 has been reported to decrease the mobilization of viable circulating endothelial cells and putative endothelial progenitor cells [27].

As an indirect inhibitor, TSP-1 binds and modulates the activity/bioavailability of different mediators of angiogenesis, such as angiogenic factors, cytokines and proteases. The interaction of TSP-1 with the angiogenic factors FGF-2, VEGF, HGF, PDGF and the viral protein Tat [28-32], might be viewed as a general mechanism of angiogenesis regulation and thus a paradigm for designing therapeutic intervention.

Different therapeutic approaches have been proposed to exploit the antiangiogenic properties of TSP-1 (reviewed in [33-35]). Increased levels of TSP-1 have been obtained by gene therapy approaches, based on viral and non-viral vectors, designed to grant the constant delivery of the antiangiogenic factors necessary for an effective control of angiogenesis [36,37]. Alternatively, different classes of antiangiogenic compounds stimulate TSP-1 production, including fenofibrate, thrichostatin-A, retinoic acid, inhibitors of DNA methyltransferases and histone deacetylases (see [33,34] and references therein). Interestingly, an increase in TSP-1 production has been indicated as the main mechanism of the antiangiogenic activity of metronomic chemotherapy, the frequent administration of low dose chemotherapy proposed to optimize the antiangiogenic property of chemotherapeutics [38,39].

Different antiangiogenic sequences have been identified in TSP-1, each offering a potential tool for therapeutic exploitation [34,35]. Synthetic peptides based on the active sequences of TSP-1 have antiangiogenic and antineoplastic activity. Chemical modifications - such as use of non-natural amino-acids, cyclization, linkage to proteins, retro-inverso analogues, or polysucrose conjugates - can be successfully used to increase peptide stability, bioavailability, potency, tumor targeting ability, and to improve pharmacokinetics/pharmacodinamics (PK/PD) [23,40-42]. Two TSP-1-based peptido-mimetics developed as antiangiogenic agents for antineoplastic therapy, have reached clinical trials, ABT510 and CVX-045. ABT-510 is a modified peptide, based on the 7-mer active sequence GVITRIR present in the second type I repeat of TSP-1 [41]. It induced endothelial cell apoptosis in vitro and inhibited angiogenesis in in vivo assays [43]. ABT-510 and the related peptide ABT-526 inhibited tumor growth and reduced microvessel density in preclinical tumor models [43-45], and were particularly active in combination with chemotherapy [46,47] or other antiangiogenic agents [48]. CVX-045 is a fusion molecule, produced by covalently attaching two TSP-1 mimetic nonamer peptides to the Fab binding site of a humanized scaffold antibody, which endows the molecule with the advantageous PK of antibodies. CVX-045 has been reported active in xenograft tumor models, both as single treatment and in combination regimens [49].

## EXPLOITING PROTEIN-PROTEIN INTERACTIONS IN DRUG DISCOVERY

The TSP-1/FGF-2 interaction and its exploitation in the design of inhibitor peptide sequences represents a paradigmatic example of how the knowledge of naturally occurring protein-protein interactions can be exploited in the discovery of new drug-like inhibitors.

Indeed, protein-protein interactions (PPIs) play fundamental roles in all biological processes, and underlie a wide variety of complex pathways fundamental for either the correct functioning of cells or the development of pathological conditions [50]. As a consequence, it is not surprising that over the last few years the study and targeting of protein-protein interactions has attracted ever growing attention [51].

The development and success of structural and functional genomics initiatives combined to the improvements in proteomics and bioinformatics have provided a wealth of structural and functional information on PPI's [52-54]. This information can now be used to discover small molecules that mimic the functions of one of the two binding partners, thus acting as antagonists or regulators in pathways where a certain PPI is particularly relevant.

However, targeting protein-protein interaction interfaces has proven to be a challenging task in drug discovery [55]. The contact surfaces may indeed be large and rather flat, compared to the well-defined cavities that are typically exploited in targeting enzyme active sites in classical drug discovery efforts. Moreover, in many cases, most of the contacts defining the surfaces involve aminoacids that are not contiguous in the primary sequence. Consequently, peptides derived from short sequences may represent poor starting points from the medicinal chemistry point of view. In the same frame of thought, in most cases, there is no natural small molecule partner that either mediates or takes part in protein-protein interactions. Thus, in contrast to the development of enzyme inhibitors, there is no small molecule substrate or ligand to use as a starting point for modifications or mimics-development.

Despite all these hurdles, several success stories have emerged that provide confidence in the quest for small molecule inhibitors of PPI's.

Great impulse to this has come from the availability of a number of high resolution X-ray structures of protein-protein complexes and from the study of the effects of complex formation on the conformational properties of the partners from NMR experiments [56,57]. The combination of this structural information with the results of mutational studies has helped identify small-subsets of residues contributing most of the free energy of complex stabilization. The chemical properties and positioning of these “hot spots” can then be exploited as templates for the design of new small-molecules, as targets for fragment based drug development or for focusing high throughput screening campaigns.

The analysis of the properties of protein-protein complexes, combined to mutational scanning has led to the development of inhibitors of the cytokine Interleukin-2 [58] binding to its receptor. Another example is represented by the members of the B-cell lymphoma 2 (Bcl-2) family. These proteins, indicated as Bcl-X_L_ are important regulators of apoptosis. They form homodimers or heterodimers with other members of the family generating antiapoptotic complexes, important in tumor development and progression [59]. Analyzing the binding of these proteins to specific helical regions of BAK of BAD, it was possible to develop and design small molecules able to selectively disrupt the target protein-protein interactions with important anticancer activities [60]. Similar approaches have been applied to identify new inhibitors of the p53-HDM2 interactions or disruptors of TNF dimers [61].

An important development for the discovery of new PPI inhibitors may stem from the realization that interacting surfaces are not static. Rather, they are highly dynamic. Computational methods, mainly based on all-atom Molecular Dynamics simulations, now offer unprecedented possibilities to design molecules and pharmacophore models that target flexible receptors, taking multiple different conformational states available on the protein's energy landscape. Including an atomic level resolution description of the adaptation of the two binding partners to each other and/or differences in the solution behavior compared to the X-ray situation in the small molecule selection process has the potential to extend the chemical space of PPI inhibitors suggesting new structures, chemotypes and ultimately drugs [62-64].

Computational studies have to be validated experimentally to be really helpful in the context of rational drug design. NMR has recently emerged as a high-throughput experimental technique in drug discovery, in determining possible binding affinities and in characterizing the regions of interaction. NMR analysis helps selecting and filtering only those conformations that verify specific structural constraints obtained in solution at equilibrium, recapitulating ensemble properties that are specific only to selected molecular configurations. In particular the recent development of new exchange-based methods makes NMR spectroscopy a unique tool for accessing huge and heterogeneous systems reducing or eliminating the molecular weight limitations for receptors in interaction studies. These methods, giving exchange-transferred structural information at high sensitivity on receptor-bound ligands from unbound-state resonances, are very useful to study the interactions of small molecules or proteins with very large systems, with no need of labeling [65-67].

Overall, these concepts provide a solid basis to integrate structural, dynamic, biochemical and molecular biology information explicitly in the drug discovery process. In this context, the molecular information on TSP-1/FGF-2 binding has been exploited to discover new inhibitors of angiogenesis.

## EXPLOITATION OF THE FGF-2-BINDING SEQUENCE OF TSP-1 FOR THE DESIGN OF ANTIANGIOGENIC COMPOUNDS

**Figure 3:**
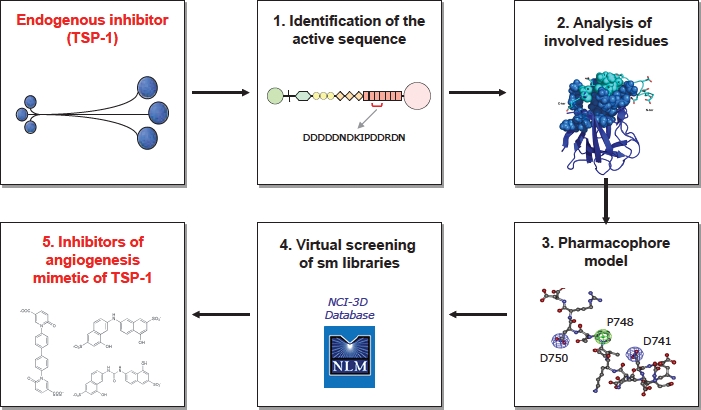
Schematic representation of the approach undertaken to develop non peptidic, small molecule inhibitors of angiogenesis mimetic of TSP-1

As mentioned above, a common aspect of a number of endogenous inhibitors of angiogenesis, including TSP-1, is the ability to bind and sequester angiogenic factors. Our work hypothesis was therefore to characterize and exploit this property of TSP-1 to design inhibitors of angiogenesis (Figure 3).

TSP-1 binds FGF-2, with high affinity (in the nanomolar range), similar to the affinity of the growth factor for heparin [29,31]. Consequently to this interaction, TSP-1 prevents FGF-2 binding to HSPG in the extracellular matrix and on the surface of endothelial cells [30], inhibiting FGF-2-mediated pro-angiogenic activation of endothelial cells, and depleting the extracellular matrix of stored FGF-2, an important component in the process of neovascularization [29,30].

In order to exploit the FGF-2-binding region of TSP-1, we first needed to identify the FGF-2-binding sequence. We therefore designed an approach allowing the identification of progressively smaller FGF-2-biding site in TSP-1. Thrombin-generated, proteolytic fragments of TSP-1 were first used, and showed that the FGF-2-binding ability was retained by the 140 kDa carboxy-terminal domain [29,30]. Using recombinant portions of TSP-1, we identified a previously undescribed antiangiogenic site in the type III repeats of TSP-1, and demonstrated that binding of the angiogenic factor FGF-2 to this site inhibits angiogenesis by sequestration of FGF-2 [31]. Finally, the peptide array technology allowed us to identify three potentially active linear sequences involved in the interaction of FGF-2 with the type III repeats of TSP-1 [68]. Binding experiments and SPR analysis with synthetic peptides corresponding to these sequences indicated that one of them, DDDDDNDKIPDDRDN (residues 739-753; named DD15) indeed bound FGF-2 with a Kd of 28.0 μM. In agreement with previous findings showing that TSP-1 impaired FGF-2 interaction with heparin, molecular dynamics (MD) analysis of the DD15/FGF-2 complex indicated that the peptide adopted an extended conformation over FGF-2 binding surface, occupying the heparin-binding site, with favorable electrostatic couplings between the Lys sidechains on the surface of FGF-2 and the negatively charged Asp groups of DD15.

Once a bioactive protein sequence is identified, two main approaches can be undertaken to develop active mimicking compounds: i) the design of natural or modified synthetic peptides or ii) the development of non-peptidic mimetic small molecules. In our case, the former option was hindered by the poor affinity of the peptide for FGF-2, and by the general poor pharmacological properties of peptides. We thus decided to undertake a search for non-peptidic, small molecule mimetic of TSP-1, using a pharmacophore-based approach that allows a high-throughput search of molecules presenting the correct spatial geometry of the functional groups required for target recognition. The power of this approach is the possibility of selecting chemically different compounds having the same target recognition and biological activity, therefore offering broad possibilities of lead selection for future pharmacological development.

The information independently obtained by MD simulations and experimental NMR data was combined to provide a cross-validated and cross-filtered atomic resolution model of the complementary interactions within the binding site. The results of extensive all-atom MD simulations provided a statistically weighted description of the most relevant interactions defining binding. It is worth noting that this MD-based approach was developed to analyze the complex interface while taking account of conformational dynamics and motional flexibility of both binding partners and focusing in particular on hydrogen-bonding, hydrophobic/aromatic, and charge-charge interaction [68]. NMR analysis, based on Saturation Transfer Difference (STD) experiments [65], identified and confirmed the identity of the peptide residues making interaction with FGF-2. Altogether, MD and NMR analyses yielded a consistent identification of DD15 residues mostly involved in the recognition of FGF-2 and stabilization of the complex, indicating that both hydrophobic and electrostatic contributions are relevant to the interaction.

**Figure 4:**
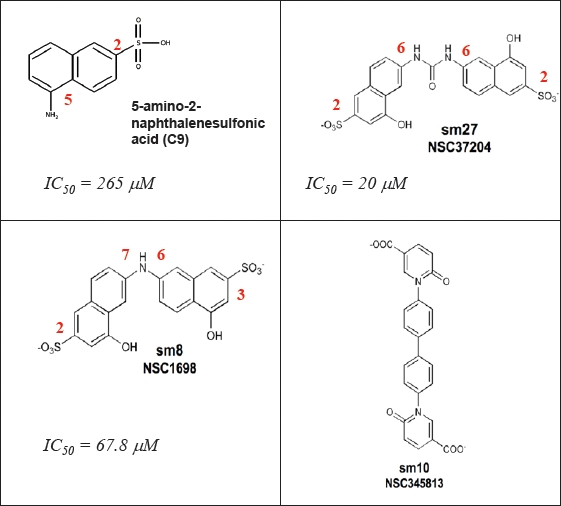
Chemical structures of sodium 5-amino-2-naphthelenesulfonate (C9, [69]) and of the active (sm8 and sm27) and less active (sm10) leads identified [68] Carbon positions of the relevant substitutions in the aromatic ring are labeled in red.

After analyzing the MD trajectories, the groups of DD15 mostly involved in stabilizing the complex with FGF-2 and their average relative positioning were identified and this information translated into a pharmacophore model that recapitulated the minimal functional and stereochemical requirements needed by a molecule to bind FGF-2. In particular, three pharmacophoric points were considered: two negative ionizable functionalities mapped over the carboxy groups of Asp741 and Asp750, and one five- or six-membered ring moiety mapped on the position of the corresponding ring of Pro748, to mimic the peptide hydrophobic patch defined by Ile747-Pro748 and provide structural rigidity to hits. The pharmacophore was used to screen the NCI2003 database of molecules (Developmental Therapeutics Program NCI/NIH). This search yielded 42 molecules. Nineteen of them, made available from the NCI (Rockville, MD) were subjected to experimental analysis of FGF-2 binding. Three molecules (named sm8, sm27, and sm10, Figure 4) were indeed able to bind FGF-2 with Kd ranging from 0.4 to 34 μM). Importantly, the small molecules retained the antiangiogenic activity of the entire TSP-1 and the type III repeats, as they prevented the binding of FGF-2 to endothelial cells and inhibited FGF-2-induced endothelial cell proliferation. Moreover, the most active molecule, sm27, inhibited FGF-2-induced angiogenesis in the chicken chorioallantoic membrane assay[68].

Interestingly these molecules met the stereochemical rules, obtained through a totally different approach, proposed to develop naphthalene sulfonates as FGF inhibitors [69]. In an attempt to identify new naphthalene derivatives that combine the highest inhibitory activity with the lowest toxicity, Fernandez-Tornero and coworkers explored a wide window of charge, size and relative position of substituents of the naphthalene ring This study allowed formulating the following stereochemical rules that may constitute the basis for the development of new antiangiogenesis treatments: 1) the sulfonate group should preferentially be located at position 2 rather than 1 of the planar aromatic ring of naphthalene; 2) derivatives containing one amino group at positions 5 or 6 are better inhibitors of aFGF mitogenic activity; 3) the size of the functional group at position 5 or 6 seems very relevant, because a significant impairment was evident when a bulky amide is introduced

It is interesting to make a comparative analysis between the structure/inhibition relationship of the most active compound found by Fernandez-Tornero and coworkers, the sodium 5-amino-2-naphthelenesulfonic acid (C9) and those of the most active (sm8 and sm27) and the less active (sm10) leads identified by our group [68] (Figure 4). The half-maximum inhibitory activity of sm27 (IC50 = 20 μM ) is more than one order of magnitude lower than that of C9 (265 μM). Indeed, both sulfonate groups are located at position 2 and both amino groups at position 6, in good agreement with the stereochemical rules formulated. The half-maximum inhibitory activity of sm8 (IC50 = 67.8 μM) is four times lower than that of C9, but three times higher than that of sm27. These features might be rationalized considering that, even if the size and functional groups are exactly the same, only one sulfonate group is located at the right position 2 in sm8 (the other one being at the wrong position 3) and only one amino group is located at the right position 6, while the other one is at the wrong position 7. Finally sm10, which does not respect at all the mentioned stereochemical rules, is endowed with modest inhibitory effect.

Our approach has allowed the identification of active small molecules mimetic of TSP-1. These leads cannot yet be considered real drug candidates, and additional efforts are required to further derivatize the leads through a combination of combinatorial chemistry, molecular dynamics and docking approaches guided by NMR experimental restraints, to improve on their drug-like properties and FGF-2 targeting affinity and specificity. The latter issue is particularly important, since the possibility exists that small molecules might partially lose the high specificity typical of peptides and eventually interact with additional targets originally not recognized by the peptide.

We believe that our study has confirmed the reliability of the pharmacophore-based strategy to develop active compounds mimetic of endogenous inhibitors for pharmacologic use. Other studies integrating 3D structural and the screening of libraries of natural products led to the discovery of a new chemical class of FGF inhibitors. Combining screening, X-ray structure determination and NMR studies to cell biology methods, proliferation assays and angiogenesis assays Gimenez-Gallego and coworkers [14] were able to identify Gentisic acid derivative as possible starting points for the development of angiogenesis inhibitors with promising therapeutic properties in vivo.

## CONCLUSIONS

The identification of TSP-1-mimetic antiangiogenic leads support the hypothesis that integrated multidisciplinary approaches can be successfully used to develop non-peptidic small molecule mimics of endogenous proteins as therapeutic agents. These leads represent a starting point for the future development of antiangiogenic agents.

This study opens new themes of investigations. Heparin recognition is a common feature of angiogenic factors. It is therefore conceivable that TSP-1, its active sequence DD15 and the identified small molecule sm27 would bind and inhibit the activity of other angiogenic factors. Should this be the case, a compound similar to sm27 might be envisioned as a general recognition backbone for heparin-binding angiogenic factors, whereas further chemical modification might be designed to tune its selectivity for specific angiogenic factors. In this respect, it is worth noting that the structure of sm27 closely mirror that of Surfen [70]. The positively charged groups of Surfen which substitute the negatively groups of sm27, confers Surfen with the ability to bind to HSPG blocking their interactions with FGF-2. This indicates that the common backbone shared by Surfen and sm27 might indeed meet the correct spacial requirements to interfere with protein/HSPG interaction.

Another potential area of research is the analysis of sequences similar to DD15 present in other proteins, including members of the TSP family as well as proteins unrelated to TSPs. This might lead to the recognition of other proteins or protein fragments able to interact with angiogenic factors, and possibly to the identification of a class of inhibitors sharing a similar structure/activity. Moreover, since FGF-2 is not only an angiogenic factor, but also a major promoter of tumor cell malignant behavior, the possibility exists that TSP-1, DD15 and sm27 have a direct anti-invasive and anti-metastatic activity, that warrants further investigation.

Finally, we think it is important to underline that our data, together with the results from other groups, demonstrate that exploiting the new knowledge on PPIs important for the regulation of angiogenesis, the integration of different levels of information ranging from the functional level to the dynamic determinants of specific molecular recognition events may ultimately spur the discovery of new drug classes active on challenging targets, overcoming the limitations and difficulties of classical drug design efforts.
